# Multi-dimensional omics characterization in glioblastoma identifies the purity-associated pattern and prognostic gene signatures

**DOI:** 10.1186/s12935-020-1116-3

**Published:** 2020-01-31

**Authors:** Yi Xiong, Zujian Xiong, Hang Cao, Chang Li, Siyi Wanggou, Xuejun Li

**Affiliations:** 1 Department of Neurosurgery, Xiangya Hospital, Central South University, 87 Xiangya RD, Kaifu District, Changsha, 410008 China; 2 Hunan International Scientific and Technological Cooperation Base of Brain Tumor Research, Xiangya Hospital, Central South University, Changsha, 410008 Hunan China

**Keywords:** Tumor purity, Glioblastoma, Tumor immunity, Tumor heterogeneity, Tumor microenvironment

## Abstract

**Background:**

The presence of tumor-associated stroma and tumor-infiltrated immune cells have been largely reported across glioblastomas. Tumor purity, defined as the proportion of tumor cells in the tumor, was associated with the genomic and clinicopathologic features of the tumor and may alter the interpretation of glioblastoma biology.

**Methods:**

We use an integrative approach to infer tumor purity based on multi-omic data and comprehensively evaluate the impact of tumor purity on glioblastoma (GBM) prognosis, genomic profiling, and the immune microenvironment in the Cancer Genome Atlas Consortium (TCGA) cohort.

**Results:**

We found that low tumor purity was significantly associated with reduced survival time. Additionally, we established a purity-relevant 5-gene signature that was an independent prognostic biomarker and validated it in the TCGA, CGGA and GSE4412 cohort. Moreover, we correlated tumor purity with genomic characteristics and tumor microenvironment. We identified that gamma delta T cells in glioblastoma microenvironment were positively correlated with purity and served as a marker for favorable prognosis, which was validated in both TCGA and CGGA dataset.

**Conclusions:**

We observe the potential confounding effects of tumor purity on GBM clinical and molecular information interpretation. GBM microenvironment could be purity-dependent, which provides new insights into the clinical implications of glioblastoma.

## Background

Glioblastoma (GBM), Grade IV glioma, is an incurable CNS malignancy of adults with high heterogeneity. Despite advances in surgery, radiotherapy, and chemotherapy, the prognosis of GBM patients has not improved significantly, and the median survival remains around 15 months [1]. Recently, increasing evidence has shown that tumor microenvironment plays a pivotal role in tumor biology, including tumor progression and drug resistance [2, 3]. The presence of specific immune infiltrates or the absence of immunosuppressive signaling were found to indicate positive prognostic features [4]. Much emphasis was placed on tumor-associated macrophages (TAMs), which could participate in tumor progression and metastasis, and influence response to chemotherapy or radiotherapy [5, 6].

Tumor purity is defined as the proportion of tumor cells in the tumor tissue. Over the past few years, tumor purity is routinely determined by pathologists through visual inspections such as immunohistochemistry (IHC) staining, which could be affected by the sensitivity of histopathology, interobserver bias, and variability in accuracy [7]. Several alternative purity estimation methods by computational approaches were developed recently, and they were based on transcriptome data, DNA methylation data or genome data [8–10]. However, purity estimates inferred by one certain omics data still confines the interpretation of purity in tumor biology systemically in previous studies [11, 12]. To overcome this, a recent study proposed a computational method for calculating the value of purity, namely, the consensus purity estimation (CPE), which was based on ABSOLUTE, ESTIMATE, LUMP and IHC methods [13].

**Fig. 1:**
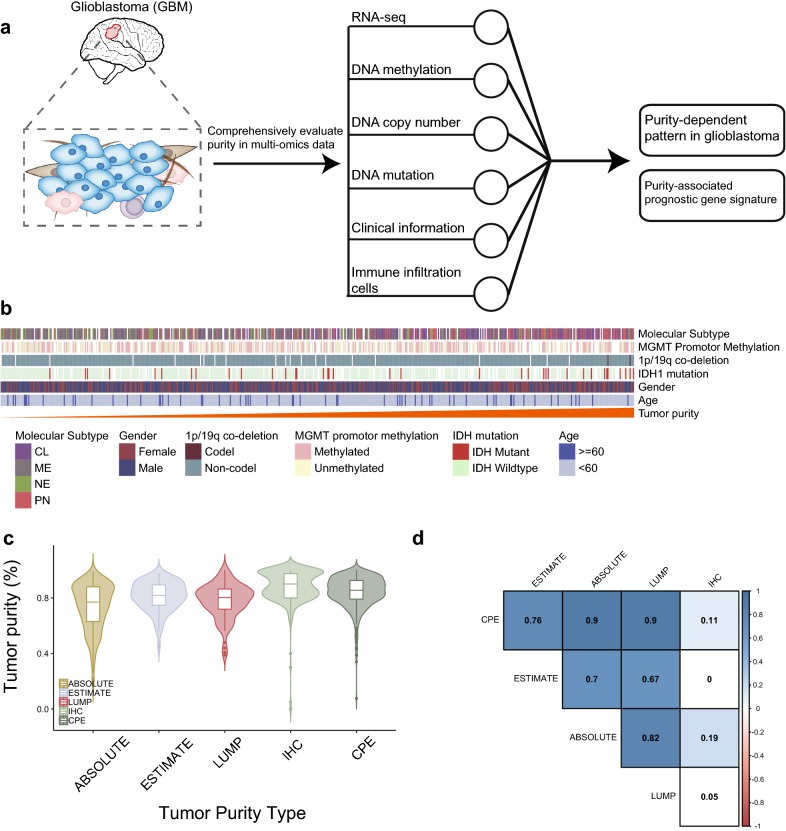
**a** The workflow of this study. **b** Heatmap of clinical and molecular characteristics of glioblastoma patients in TCGA-GBM cohort (n = 583). **c** The data distribution of tumor purity estimates. **d** Correlations (Spearman’s Rho) between tumor purity estimates inferred by different methods

Despite these discoveries, however, little is known regarding the association between the purity and the genomic or clinicopathological features in glioblastoma. Besides, the relationship between purity and glioblastoma microenvironment remains unclear. In this study, we employed the CPE method to estimate the tumor purity and sought to identify the potential confounding effects between tumor purity and clinical or molecular characteristics (Fig. 1a). Thus, we investigated the correlation between tumor purity and genomic alterations, biological pathways as well as immune cell compositions in the microenvironment, which could deepen our understanding of glioblastoma biology and provide new insights into the clinical management of glioblastoma.

## Materials and methods

### Datasets and data processing

A total of 583 patients with glioblastoma from the TCGA cohort were enrolled in this study. Clinical information and data of molecular biomarkers were acquired from TCGA publications as previously described [14]. In CGGA (Chinese Glioma Genome Atlas) cohort, IlluminaHiSeq RNA-seq data and clinical and molecular information from 144 patients with glioblastoma were obtained via the CGGA database (http://www.cgga.org.cn). The raw data for the GSE4412 dataset and corresponding clinical data were acquired from the Gene Expression Omnibus (https://www.ncbi.nlm.nih.gov/geo/query/acc.cgi?acc=GSE4412).

### Tumor purity analysis

Tumor purity scores were inferred by the consensus purity estimation (CPE) method as previously described [13]. The tumor purity score was derived from the median value estimated from ABSOLUTE, ESTIMATE, LUMP and IHC methods after normalization [9, 10]. To prevent confusion, we defined the CPE score as purity score and used it throughout the article, unless specifically noted.

### Transcriptomic data analysis

TCGA AffyU133a gene expression array data and IlluminaHiSeq RNA-seq data of GBMs were downloaded from https://tcga-data.nci.nih.gov/ via Xena Browser developed by UCSC. Statistical ranking for purity scores by the top and bottom quartiles were defined as high-purity and low-purity, respectively. Differentially expressed genes (DEGs) between high-purity and low-purity samples were identified using the R/DESeq 2 package or R/Limma package. DEGs with false Discovery Rate (FDR) < 0.05 and fold change > 2 (or < 0.5) were included in further analysis. Both the enrichment analysis and GSEA were performed using R package *clusterProfiler* and *ReactomePA* [15–17]. GSVA analysis of hallmark pathways and metabolic pathways was implemented as previously described [18] by R/GSVA package [19]. GO Enrichment network was drawn using EnrichmentMap software [20] for interpretation.

### Methylation analysis

Illumina Infinium DNA methylation platform arrays HumanMethylation450 data in the TCGA-GBM cohort were downloaded from https://tcga-data.nci.nih.gov/ via Xena Browser developed by UCSC. Data were separated into different datasets according to purity. Data were further normalized and processed by using *ChAMP* package with default parameters [21].

### Survival analysis

R package *survival* and *survminer* were used for overall survival analysis. Cox proportional hazard (PH) model was executed by Coxph and Survfit functions from R packages. The Kaplan–Meier curves were employed to estimate overall survival distribution.

### Genomics analysis, intra-tumor heterogeneity (ITH) analysis

Somatic copy number alterations (SCNA) data (minus germline CNV) were downloaded from GDAC Firehose and separated into different datasets according to the purity. SCNA events were detected by GISTIC2.0 using the segmented Affymetrix SNP 6.0 microarray data [22]. Somatic variants files of GBMs in MAF format were downloaded from https://tcga-data.nci.nih.gov/ via Xena Browser developed by UCSC and downstream analysis was performed by R/maftools package [23]. For somatic nucleotide variations (SNVs), we calculated the total mutation count. We used Mutant-allele tumor heterogeneity (MATH) as a quantitative estimate of intra-tumor heterogeneity (ITH) [24]. Subclone numbers within each sample were inferred by *pyclone* software as previously described and were normalized with tumor purity [25].

### Immune cellular fraction estimates

The relative fractions of 24 immune cell types within the leukocyte compartment were estimated using gene sets introduced by Gabriela et al. [26]. IlluminaHiSeq RNA-seq raw counts across all genes for each sample were divided by the gene’s maximum transcript length to represent a coverage depth estimate, which was then rescaled to sum to a total depth of 1e6 and thus can be interpreted as transcripts per million (TPM) [27]. We used the RNA-seq TPM data as input and the enrichment of an immune cell type meta-gene in a given sample were then scored using single-sample gene set enrichment (ssGSEA) analysis [28], as implemented in the GSVA R package [19], with subsequent z-scoring across samples. Note that these enrichments should not be interpreted as deconvolutions of actual cell-type proportions.

### Statistics analysis

All statistical analyses were performed using R software, version 3.5.1 (The R Foundation for Statistical Computing, http://www.rproject.org/). Continues variables between groups were compared by the Student’s *t* test, one-way analysis of variance (ANOVA) test or the Wilcoxon rank-sum test. Correlations between continuous variables were evaluated by Spearman or Pearson correlation analyses. For all statistical analyses, the P value of 0.05 was taken as the significant threshold in all tests.

## Results

### Tumor purity and clinicopathological and molecular features

An overview of the analytical strategy here is shown in Fig. 1a. Tumor purity scores were calculated by consensus purity estimation (CPE) method based on ABSOLUTE, ESTIMATE, LUMP and IHC algorithms (Additional file 1: Table S1). The purity inferred by CPE is the normalized purity scores inferred from the above four methods (Fig. 1b, c). We observed that tumor purity inferred by the CPE method was significantly positively correlated with purity calculated based on ABSOLUTE, LUMP, ESTIMATE (Spearman’s correlation, rho = 0.90, 0.90, 0.76, respectively) (Fig. 1d), suggesting the rationality of this method.

**Fig. 2:**
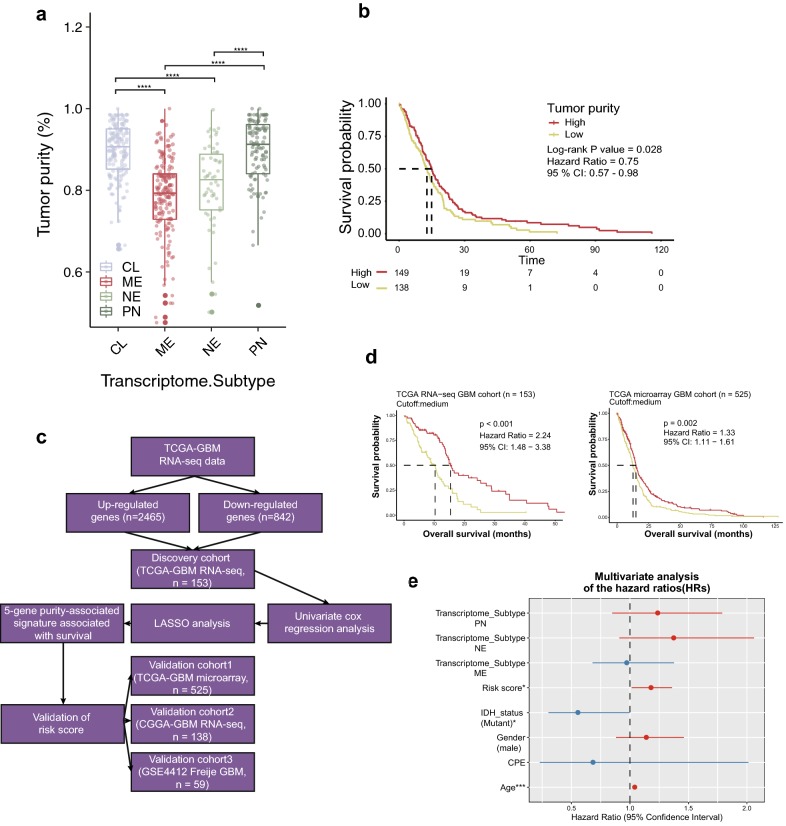
**a** Boxplots showing comparisons between tumor purity (CPE scores) between transcriptome molecular subtypes. For each comparison, data were analyzed using student’s t-test or one-way ANOVA. Box plot center, box, and whiskers correspond to the median, IQR and 1.5xIQR (interquartile range), respectively. **b** Kaplan–Meier curves for overall survival according to tumor purity. **c** Workflow of construction of 5-gene purity-associated signature. **d** Kaplan–Meier curves for overall survival devided by risk score in TCGA-GBM dataset. **e** Risk score is an independent prognostic factor in TCGA-GBM dataset. *CL* classical, *ME* mesenchymal, *NE* neural, *PN* proneural

We next identified the relationship between tumor purity and clinicopathological/molecular features in the TCGA-GBM cohort (Fig. 1b). We observed that tumor purity was significantly enriched in IDH-mutant samples or MGMT-promoter-methylated samples (student’s t-test, P < 0.001, P = 0.024, respectively) (Additional file 2: Fig. S1), which were associated with favorable prognosis. Meanwhile, we analyzed the purity distribution among four GBM molecular subtypes, namely proneural, classical, mesenchymal and neural, based on transcriptome profile [29]. We found that decreased tumor purity levels were enriched in neural or mesenchymal molecular subtypes, which were generally connected with the malignant progression of glioma (Fig. 2a). These findings emphasized that purity was closely related to specific clinicopathological/molecular features.

### The prognostic role of tumor purity in glioblastoma

To illustrate the correlation between purity and overall survival, patients were divided into three groups according to the quantiles of the purity score. Kaplan–Meier curve showed that low purity GBM samples (tumors in the bottom 25th percentile) display significantly worse clinical outcomes (High vs Low, HR = 0.75, p = 0.028, Log-Rank Test) (Fig. 2b). Subgroup analysis revealed that the low tumor purity has dismal prognosis in the female, G-GIMP subtype, proneural subtype, and MGMT promoter methylated patients (Additional file 2: Fig. S2).

### Construction of a purity-associated gene signature using transcriptomic data

The workflow of data processing and signature construction is shown in Fig. 2c. We first take TCGA RNA-seq dataset as a training set. We divided patients into high-purity cohort (tumors in the top 25th percentile) and low-purity cohort (tumors in the bottom 25th percentile) and differentially expressed genes (DEGs) analysis were performed. By comparing low-purity samples with high-purity samples, we found that 3307 differentially expressed genes, including 2465 upregulated (highly expressed genes in low-purity samples) and 842 downregulated genes (highly expressed genes in high-purity samples). We next evaluated the prognostic impact of those genes in training set using univariate Cox regression analysis. Finally, we identified a purity-relevant 5-gene signature using the least absolute shrinkage and selection operator (LASSO) Cox regression algorithm. We calculated a risk score by integrating the z-score gene expression data and the corresponding coefficients derived from the multivariate Cox regression analysis. The risk score is as follow: risk score = 0.152*SNCB + 0.003*KCNN4 + 0.012*FCGR2C + 0.348*PLAUR + 0.067*LSP1. As a result, a significant difference in overall survival (OS) between the high-risk group and the low-risk group in the training set (HR = 2.24, 95% CI 1.48–3.38, p < 0.001) (Fig. 2d). Moreover, we validated the prognostic value of this gene signature in the TCGA-GBM microarray set, CGGA-GBM RNA-seq set and GSE4412 set (Fig. 2d, Additional file 2: Fig. S3). Finally, we performed a multivariate Cox analysis including tumor purity, gender, age, IDH mutation status, risk score as covariates. We identified that the purity-relevant gene signature was an independent prognostic indicator (HR = 1.17, 95% CI 1.01–1.36, p = 0.031) (Fig. 2e, Additional file 1: Table S2).

### Functional annotation of transcriptomic and methylation analysis in tumor purity

**Fig. 3:**
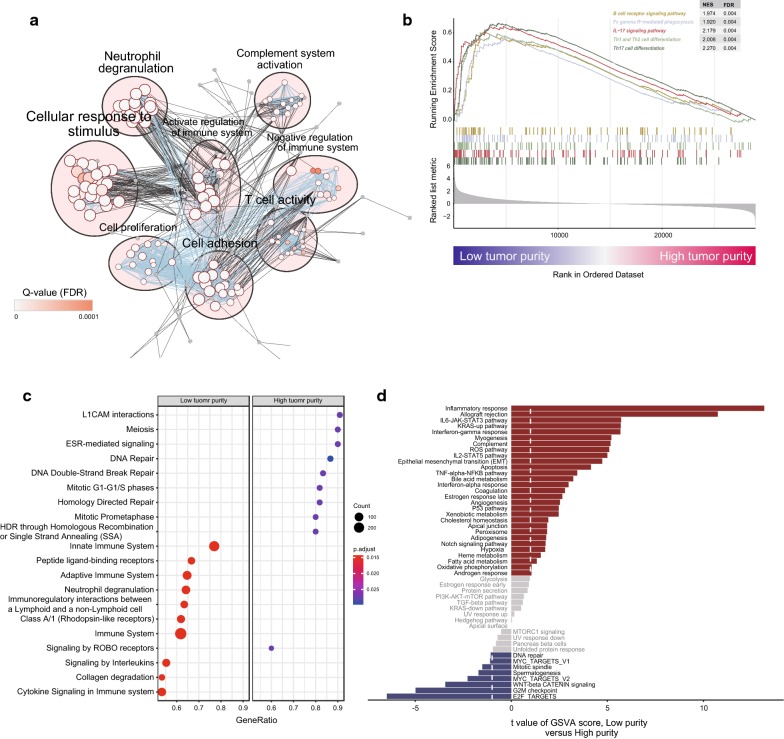
**a** GO enrichment analysis revealed enrichment of immune-related pathways in low purity samples. **b** GSEA enrichment analysis revealed enrichment of specific KEGG pathways in low purity samples. **c** Differentially enriched REACTOME pathways in samples with low tumor purity (left) or high tumor purity (right). **d** Differences in pathway activity were analyzed using GSVA and t values were shown from a linear model

We first performed unsupervised clustering of transcriptomic data based on t-SNE or PCA (principle components analysis), which could also divide patients into different groups according to purity (Additional file 2: Fig. S4). To further elucidate the mechanism underlining purity subgroups in transcriptomic architecture, we annotated DEGs using either functional enrichment pathway analysis or gene set enrichment analysis (GSEA) in the TCGA-GBM RNA-seq dataset. GO enrichment analysis of biological processes for the upregulated genes in low-purity samples revealed significant enrichment in “immune response” GO terms (Fig. 3a). Further, GSEA of the pre-ranked gene list revealed that low-purity samples were significantly enriched in immune-related pathways, including B cell receptor signaling pathway, Fc gamma R-mediated phagocytosis, and IL-17 signaling pathway (Fig. 3b). In addition, to illustrate activated reactions, pathways and biological processes in all samples, Reactome enrichment analysis was performed. As expected, samples with low-purity was significantly enriched in immune-related signaling pathways and immunoregulatory interactions whereas samples with high-purity showed significant enrichment in cell cycle regulation and DNA repair pathways (Fig. 3c).

Furthermore, we set out to identify the differences in pathway activity among two purity subgroups. We performed gene set variation analysis (GSVA) to assign pathway activity estimates to individual samples. We observed that immune-regulation pathways such as IL6-JAK-STAT3 signaling pathway, and IL2-STAT5 signaling pathway showed high pathway activity in the low-purity, whereas cell cycle regulation pathways such as G2M checkpoint signaling pathway, E2F signaling pathway displayed high pathway activity in the high-purity (Fig. 3d). All these results suggested the crucial role of regulation of the immune system in low-purity samples.

Comparing malignancy tissues with normal tissues is a common strategy to identify genes associated with tumor progression or tumor-specific markers. Thus, we used the R/Limma package to perform DEGs analysis to compare the GBM samples (from TCGA cohort) with the normal brain samples (from GTEx cohort) [30]. As purity could be a confounding factor, we controlled purity in DEGs analysis. We observed marked differences in gene expression level before and after purity adjustment and large number of genes were identified as differentially expressed genes after purity adjustment (Additional file 1: Table S3). Genes upregulated in tumor samples could be a marker for tumorigenesis. Here we detected 7460 genes that were upregulated in tumor after purity adjustment, which could be novel genes altered in tumorigenesis (Additional file 2: Fig. S5). Meanwhile, expression values of immune checkpoints genes were important markers in immunotherapy. However, we found that programmed death 1 (PD-1, encoding by *PDCD1* gene) could be upregulated in traditional DEGs analysis. However, when purity was controlled, we did not detect statistically significant results in DEGs analysis (Additional file 1: Table S4).

We also compared DNA methylation profile of GBM samples between high-purity and low-purity samples. We identified differentially methylated probes (two-sided t-test FDR < 0.05) and probes resided in gene promotor were selected as we considered DNA methylation regulation in these genes could be purity-associated. As expected, KEGG enrichment analysis showed that several immune-related pathways are involved in these differentially methylated genes, which could partially explain the differentially expressed genes in the transcriptome (Additional file 2: Fig. S6). In summary, these observations suggested the importance of considering purity as a confounding factor in transcriptome and methylatome analysis.

### Genomics alterations and tumor purity

**Fig. 4:**
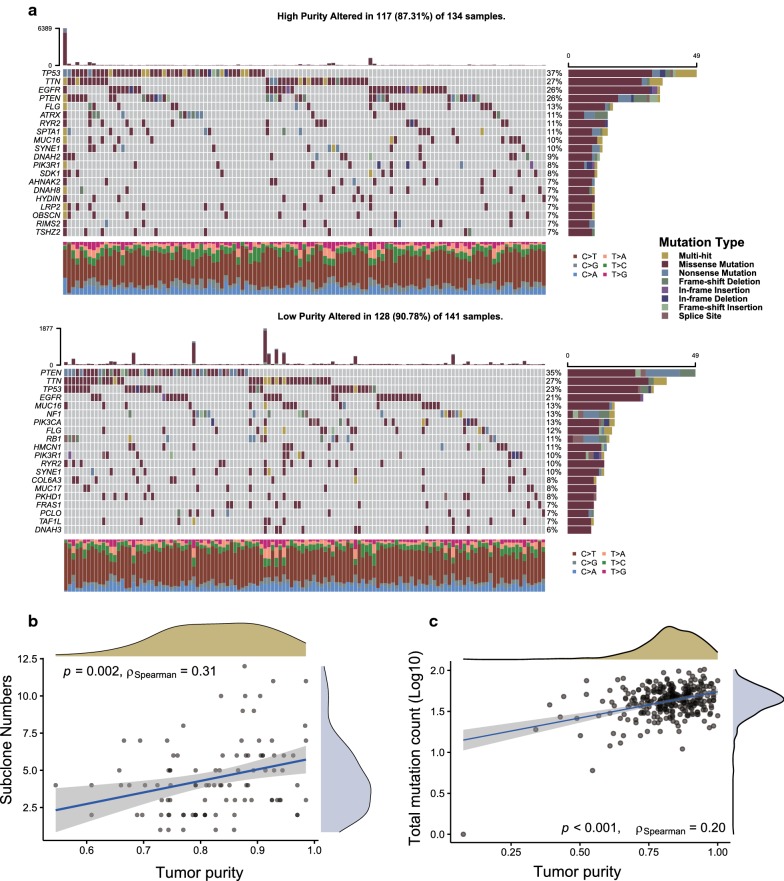
**a** Oncoprint summarizing recurrently altered genes and their distribution in TCGA-GBM high- purity samples (upper panel) or low-purity samples (lower panel). (**b**, **c**) Correlation plot showing Spearman’s Rho between purity and mutation count or subclone numbers

Genomic data from TCGA-GBM dataset were further analyzed to unveil the possible mechanisms affecting tumor purity in terms of the inter-patient genomic heterogeneity. The oncoprint showed the distinct landscape of somatic single nucleotide variants (SNVs) and indels in two tumor purity subgroups (Fig. 4a). *TP53, TTN, EGFR, PTEN* are the most frequently mutated genes in high-purity subgroups while *PTEN, TTN, TP53, EGFR* genes are the most frequently altered in the low-purity subgroup. We further explored the genomic mutations in pathways. By analyzing ten canonical oncogenic signaling pathways [31], we found the significantly high mutation frequency in cell cycle pathways for the low-purity group (P = 0.0253, Fisher’s exact test) (Additional file 1: Table S5). In addition, we observed that mutation abundance was significantly positively correlated with purity (Fig. 4b).

Next, we explored the association between SCNA and purity (Additional file 2: Fig. S7a). A large number of cytobands are either significantly amplified or deleted regardless of the influence by purity (Additional file 2: Fig. S7b). We performed GO enrichment analysis of biological processes of genes that exclusively altered in either high-purity or low-purity subgroup, which showed mainly differences in the immune regulation process (Additional file 2: Fig. S8). Furthermore, after overlaid with DEGs identified by RNA-seq, we found that 606 genes locate within aberrantly amplified regions, including 35 (5.8%) genes up-regulated in low purity subgroup, suggesting that differential expression of these genes were partially due to copy number variations.

The impact of purity on clonal architecture remains poorly investigated. We next performed a clonality analysis and calculated the MATH value to infer intra-tumoral heterogeneity (ITH). However, there is no significantly statistical difference in MATH value between the two groups (Wilcoxon rank-sum test, P = 0.414). Interestingly, we observed that decreased subclone numbers were associated with low-purity samples (Fig. 4c). We also found that high-purity was associated with high percent aneuploidy, suggesting genome instability may be enriched in the high-purity (Additional file 2: Fig. S9). Taken together, these findings confirm that purity can be a confounding factor in genomic architecture and purity was an important feature at the genomic level.

### Tumor infiltration and tumor purity

**Fig. 5:**
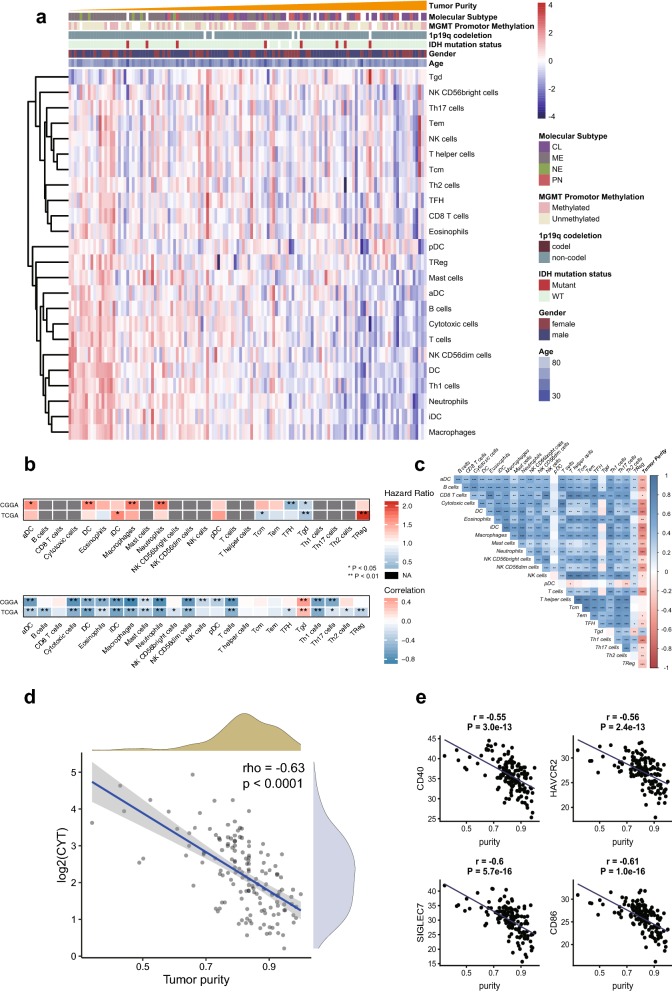
**a** The landscape of immune cell infiltrates sorted by increasing purity in TCGA-GBM RNA-seq dataset. Immune cell infiltrates were estimated by ssGSEA algorithm. **b** The correlation between the proportion of immune cell infiltrates and survival (upper panel) or purity estimates (lower panel) in TCGA-GBM or CGGA RNA-seq cohort. Purity values in CGGA cohort were inferred by ESTIMATE method. **c** Correlation plot showing Spearman’s Rho between cell types in TCGA-GBM. **d** Scatter plot of correlation of tumor purity and CYT (a geometric mean of GZMA and PRF1; y-axis in log2 scale). **e** Correlation between immune checkpoints gene expression (TCGA RNA-seq dataset) and tumor purity. Pearson’s correlation coefficients (r) are stated

To explore the tumor microenvironment of GBM, the cell abundance of tissue-infiltrating immune cells was estimated in the RNA-seq dataset from the TCGA and CGGA cohort. We estimated 24 subpopulations of immune cells by using ssGSEA strategy. As immune cells composed the majority of non-tumor components of the microenvironment, the proportion of main immune cells is inversely correlated with tumor purity (Fig. 5a, b). We also investigated the association between immune cell types and prognosis of patients (Fig. 5b). The association was varying in different cohorts. The cell types associated with worse prognosis are iDCs and Tregs in TCGA-GBM cohort while aDCs, DCs, macrophages, neutrophils correlated with worse prognosis in CGGA-GBM cohort. Interestingly, we found that gamma delta T cells (Tgd) were enriched in high-purity samples and were associated with favorable prognosis (Fig. 5b) in both TCGA and CGGA cohort (Log-Rank Test, P < 0.05). Furthermore, we observed a significant correlation between multiple cell types (Spearman’s correlation, P < 0.05) (Fig. 5c). Also, we used a simple formula to estimate immune cytolytic activity (CYT), which is assessed by a geometric mean of GZMA and PRF1 expression (TPM value). We found that CYT was significantly correlated with tumor purity (rho = − 0.63, P < 0.001) (Fig. 5d). Consistently, the cytolytic activity could also be a biomarker for unfavorable prognosis [32]. However, we did not identify a significant correlation between mutation abundance and CYT (Additional file 2: Fig. S10). Finally, we examine the impact of tumor purity on the expression of immune checkpoints genes. As expected, the expression level of *HAVCR2, CD40, SIGLEC7, CD86* genes are inversely correlated with tumor purity (Fig. 5d). Taken together, these findings demonstrated that purity was an important characteristic of tumor microenvironment.

## Discussion

To our knowledge, this is the first comprehensive study to investigate the confounding effects of tumor purity based on the CPE method in a series of clinicopathological, genomic, immune parameters for GBM patients. The key findings of this study were: 1. We identified purity-dependent distinct patterns associated with genomic and clinicopathologic features, which supports the hypothesis that tumor purity is an intrinsic characteristic of samples reflecting differences in the tumor microenvironment [13]. 2. We constructed a purity-associated gene signature which would be prognostically relevant. 3. By analyzing the GBM tumor microenvironment, we revealed that gamma delta T cells were positively associated with purity and were a favorable prognostic indicator.

Gene expression subtypes of GBM identified by unsupervised clustering have emerged as an important tool to understand GBM biology [29, 33]. We demonstrated that the low-purity was tightly associated with mesenchymal or neural subtypes. In accordance with previous studies, mesenchymal subtype was reported to be linked with tumor-associated glial and microglial cells and neural subtype was related to the tumor margin where normal neural tissue could be easily collected [33]. To analyze the confounding effect of purity based on transcriptomic data, we performed two parallel strategies. First, we assessed the DEGs between tumor and normal tissue samples, identified before and after purity adjustment, which is a well-established approach to screen tumor-associated biomarkers. We revealed that up-regulated genes identified after purity adjustment may play important roles in the biomarker setting, which requires further validation. Second, we also analyzed the upregulated and downregulated genes regarding their correlations with purity. We observed that, genes associated with high-purity, were significantly enriched in pathways related to the tumor-intrinsic characteristics such as abnormalities in cell cycle regulation and impaired DNA repair machinery; In low-purity samples, upregulated genes were commonly enriched in immunoregulation and cellular interaction pathways, suggesting a phenotype of tumor microenvironment with increased immune-infiltration in these patients. Moreover, several pathways were activated in the low-purity phenotype. For instance, in tumor immunity, IL-10 signaling pathways plays a dual role that IL-10 promotes tumor immune escape by inhibiting inflammatory cytokines, and conversely induces tumor-specific CD8^+^ T cells infiltration and promotes their cytotoxic activity [34]. In addition, IL-17-producing cells, on the other side, were found promoting tumor infiltration and acquired survival benefits in the metabolites-deficiency tumor microenvironment [35]. We also found that the IL6-JAK-STAT3 signaling pathway, which drives the proliferation, survival, invasiveness, and metastasis of tumor cells and suppresses the antitumor immune response in the tumor microenvironment [36], displayed high pathway activity by GSVA analysis in this study. Hyperactivation of this pathway is generally associated with poor prognosis and thus this pathway could be therapeutically targeted by inhibitors [36].

In previous studies, purity levels were associated with histological subtypes and histological grades, as well as survival time [11–13, 37]. Consistent with these findings, tumor purity could be a potential prognostic indicator for GBM since low purity cases were associated with poor prognosis in our study. As tumor purity could not be an independent prognostic factor while controlling other factors such as IDH mutation status, age, etc., we were motivated to establish a purity-associated gene signature. In our 5-gene signature, to take the *PLAUR* gene as an example, the *PLAUR* gene encodes the urokinase receptor (uPAR) and had the largest positive coefficient in the Cox regression model. The prognostic value of *PLAUR* gene has been reported [38]. *PLAUR* could be functionally related to tumor growth and angiogenesis [39].

In this study, we also reported that the tumor mutation burden (TMB) of the samples was positively correlated with purity. Of note, we also identified that high-purity phenotype correlates with high aneuploidy, that is, increased genome instability. The possible explanations are that high genome instability is often associated with high TMB and increased pro-inflammatory activity that causes a higher percent of tumor necrosis component with decreased immune cell infiltration [5]. Strikingly, we observed a decreased total number of subclonal numbers in low-purity samples, which is consistent with a recent study in renal cell carcinoma [40]. The possible mechanism behind this phenomenon is that during immune selection, increased immunoediting was found to eliminate tumor clones in samples with high immune infiltration [41].

We set out to analyze the immune microenvironment architecture for glioblastoma. We demonstrated that most immune cells inversely correlate with the purity whereas Tgd (T gamma delta) cells are highly infiltrated in the high purity subgroup. Consistent with the previous study [42], increasing Tgd infiltration conferred favorable prognosis in both the TCGA and CGGA cohort, which could partially help to explain favorable prognosis in the high-purity subgroup. In mechanism, gamma delta T cells have been reported with well-established protective roles in cancer, largely based on their potent cytotoxicity and interferon-γ production [43], implying a potential predictive role in GBM immunotherapy.

One of the main advantages of our research was the use of CPE to evaluate the tumor purity and systematic analysis of the purity in multidimensional profiles for GBM. Nevertheless, the present study has several limitations. First, due to the retrospective setting of the TCGA and CGGA samples, results need to be carefully assessed and validated in future prospective studies. Second, immune infiltration analysis was performed based on transcriptomic data. Thus, our analyses were limited without cell phenotype confirmation by other methods. Due to the natural complexity of glioblastoma tumor microenvironment, further studies to promote its understanding might be helpful.

## Conclusion

In summary, we systematically evaluated the role of tumor purity in the GBM prognosis, genomics, and transcriptome alterations as well as tumor immunity microenvironment. The tumor purity influences the intra-tumor heterogeneity and genomic architecture. It is important for further studies in glioblastoma biology to consider tumor purity as a confounding effect in the design.

## Supplementary information

**Additional file 1: Table S1.** The purity values in TCGA-GBM cohort. **Table S2.** Cox proportional hazards model in TCGA-GBM cohort. **Table S3.** Differentially expressed genes between tumor and normal samples. **Table S4.** Expression of Immune checkpoint molecules before and after purity adjustment. **Table S5.** Differentially mutated gene frequency in oncogenic signaling pathways.

**Additional file 2: Figure S1.** Relation between purity and IDH mutation status or MGMT promotor methylation status. **Figure S2.** The prognostic value of purity in stratified GBMs. **Figure S3.** The prognostic role of purity-associated risk score in CGGA or GSE4412 cohort. **Figure S4.** Unsupervised analyses of global transcriptional similarities and differences between two purity subgroups. **Figure S5.** Adjustment of purity in differentially expressed genes analysis. **Figure S6.** Enrichment of KEGG pathways in differentially methylated genes. **Figure S7.** Relation between purity and genomic alterations. **Figure S8.** GO enrichment analysis of differentially amplified genes or differentially deleted genes between purity subgroups. **Figure S9.** Correlation between tumor purity and genomic instability. **Figure S10.** Correlation between CYT and mutation abundance.

## Data Availability

The datasets used and/or analyzed during the current study are available from the corresponding author on reasonable request.

## Abbreviations

CGGA: Chinese Glioma Genome Atlas
CPE: consensus purity estimation
CNS: central neural system
CL: classical
CNV: copy number variation
DEG: differentially expressed gene
GBM: glioblastoma
G-CIMP: glioma CpG island methylator phenotype
GO: gene ontology
HR: hazard Ratio
ITH: intra-tumor heterogeneity
IHC: immunohistochemistry
KEGG: Kyoto Encyclopedia of Genes and Genomes
MATH: mutant-allele tumor heterogeneity
MES: mesenchymal
NE: neural
PN: proneural
PCA: principle components analysis
SCNA: somatic copy number alteration
SNV: somatic nucleotide variations
t-SNE: t-distributed stochastic neighbor embedding
TMB: tumor mutation burden
TCGA: The Cancer Genome Atlas Consortium
