# Supplementary material for: Multi-dimensional omics characterization in glioblastoma identifies the purity-associated pattern and prognostic gene signatures
Source: Cancer Cell Int. 2020 Jan 31;20:37. doi: 10.1186/s12935-020-1116-3 (PMC6995093; doi:10.1186/s12935-020-1116-3)
Supplement: Supplementary file 2 — Additional file 2: Figure S1. Relation between purity and IDH mutation status or MGMT promotor methylation status. Figure S2. The prognostic value of purity in stratified GBMs. Figure S3. The prognostic role of purity-associated risk score in CGGA or GSE4412 cohort. Figure S4. Unsupervised analyses of global transcriptional similarities and differences between two purity subgroups. Figure S5. Adjustment of purity in differentially expressed genes analysis. Figure S6. Enrichment of KEGG pathways in differentially methylated genes. Figure S7. Relation between purity and genomic alterations. Figure S8. GO enrichment analysis of differentially amplified genes or differentially deleted genes between purity subgroups. Figure S9. Correlation between tumor purity and genomic instability. Figure S10. Correlation between CYT and mutation abundance. [file 12935_2020_1116_MOESM2_ESM.pptx]

## Slide 1
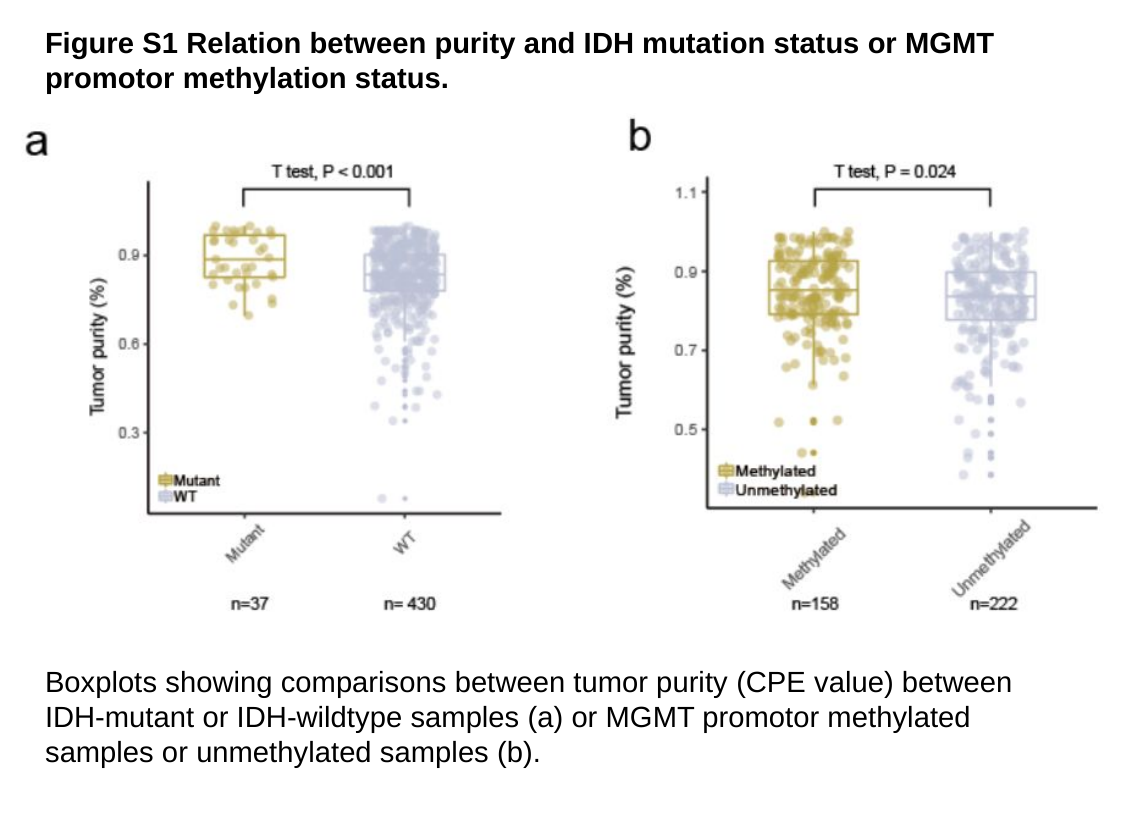

Figure S1 Relation between purity and IDH mutation status or MGMT promotor methylation status.
Boxplots showing comparisons between tumor purity (CPE value) between IDH-mutant or IDH-wildtype samples (a) or MGMT promotor methylated samples or unmethylated samples (b).

## Slide 2
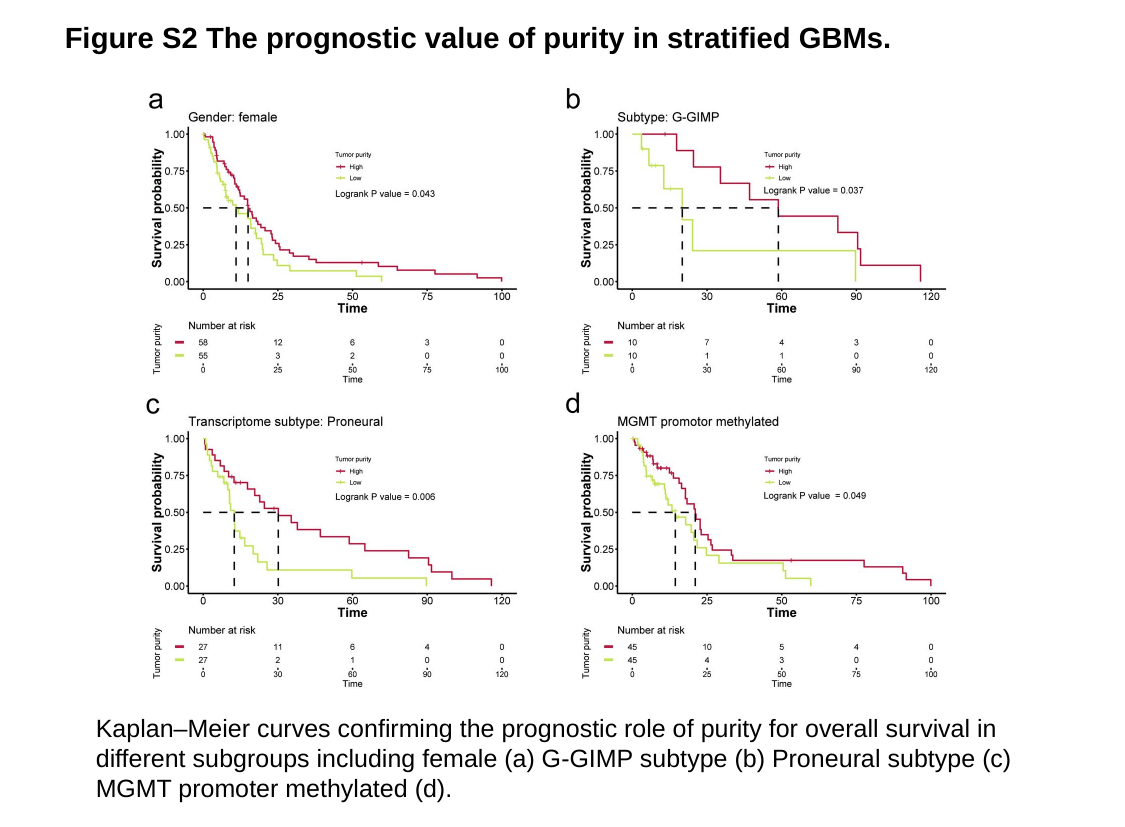

Figure S2 The prognostic value of purity in stratified GBMs.
Kaplan–Meier curves confirming the prognostic role of purity for overall survival in different subgroups including female (a) G-GIMP subtype (b) Proneural subtype (c) MGMT promoter methylated (d).

## Slide 3
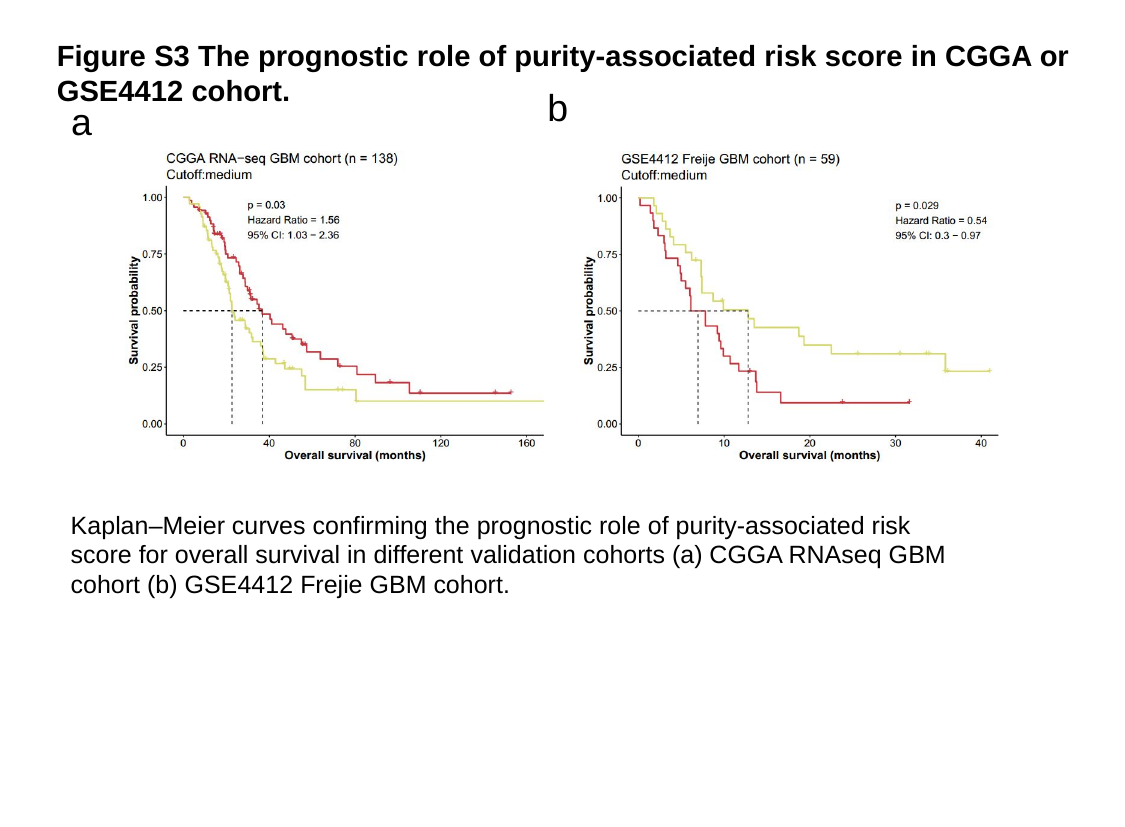

Figure S3 The prognostic role of purity-associated risk score in CGGA or GSE4412 cohort.
b
a
Kaplan–Meier curves confirming the prognostic role of purity-associated risk score for overall survival in different validation cohorts (a) CGGA RNAseq GBM cohort (b) GSE4412 Frejie GBM cohort.

## Slide 4
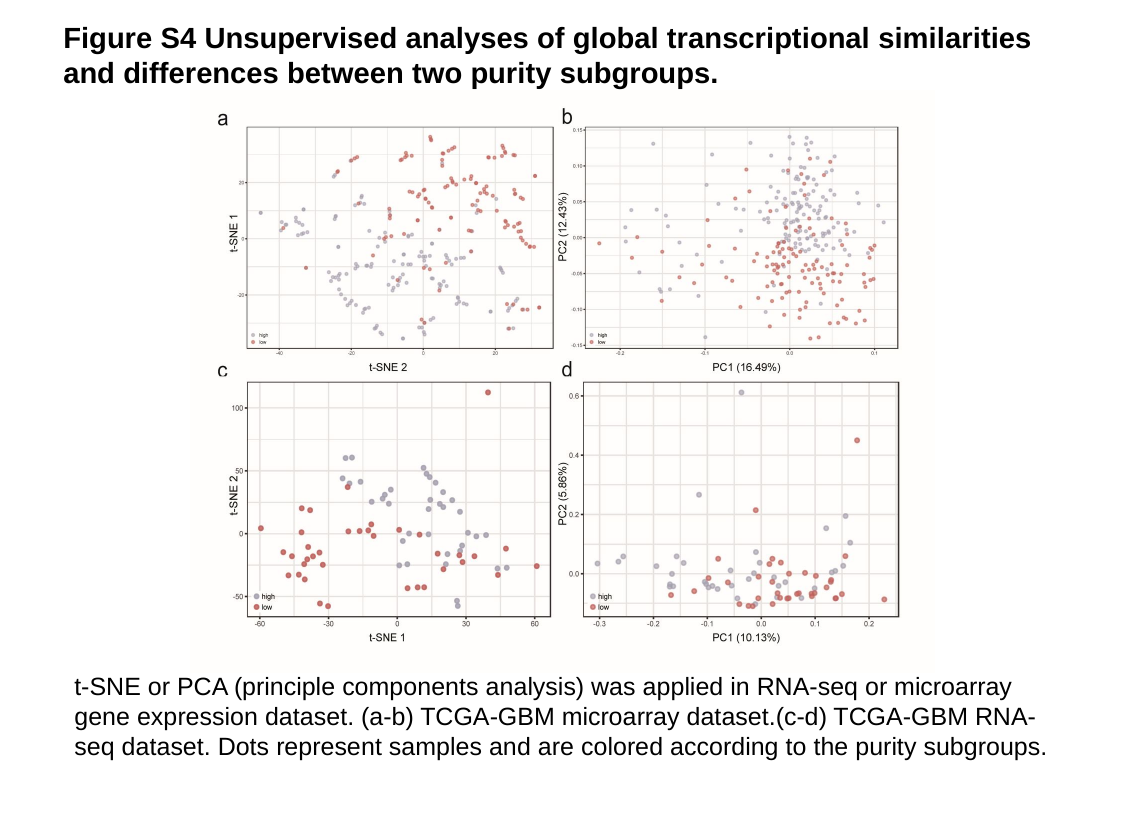

Figure S4 Unsupervised analyses of global transcriptional similarities and differences between two purity subgroups.
t-SNE or PCA (principle components analysis) was applied in RNA-seq or microarray gene expression dataset. (a-b) TCGA-GBM microarray dataset.(c-d) TCGA-GBM RNA-seq dataset. Dots represent samples and are colored according to the purity subgroups.

## Slide 5
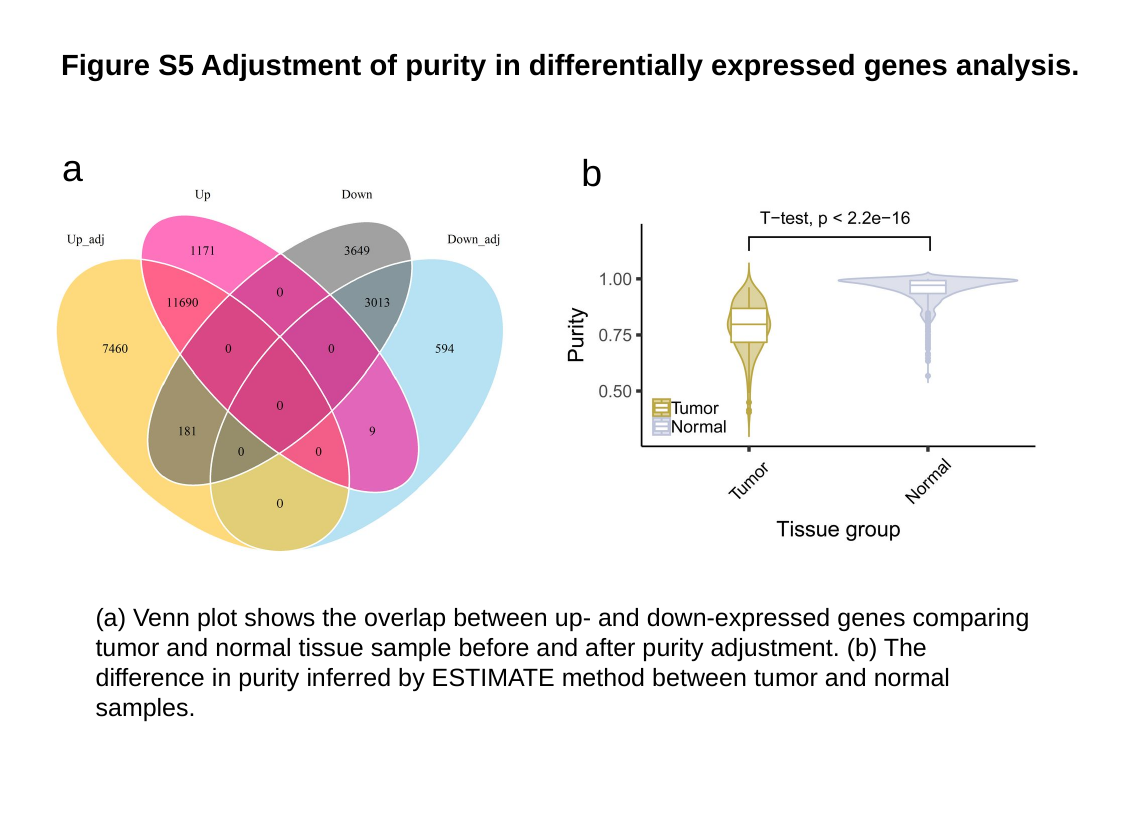

Figure S5 Adjustment of purity in differentially expressed genes analysis.
a
b
(a) Venn plot shows the overlap between up- and down-expressed genes comparing tumor and normal tissue sample before and after purity adjustment. (b) The difference in purity inferred by ESTIMATE method between tumor and normal samples.

## Slide 6
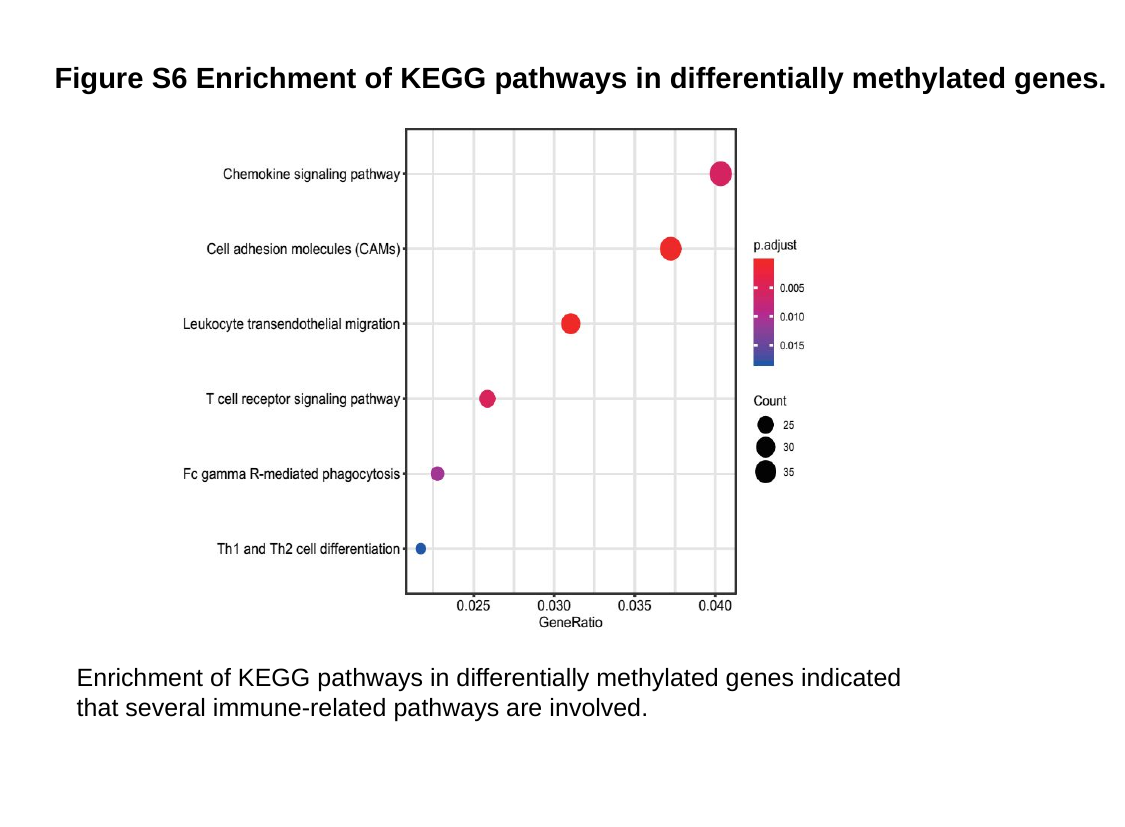

Figure S6 Enrichment of KEGG pathways in differentially methylated genes.
Enrichment of KEGG pathways in differentially methylated genes indicated that several immune-related pathways are involved.

## Slide 7
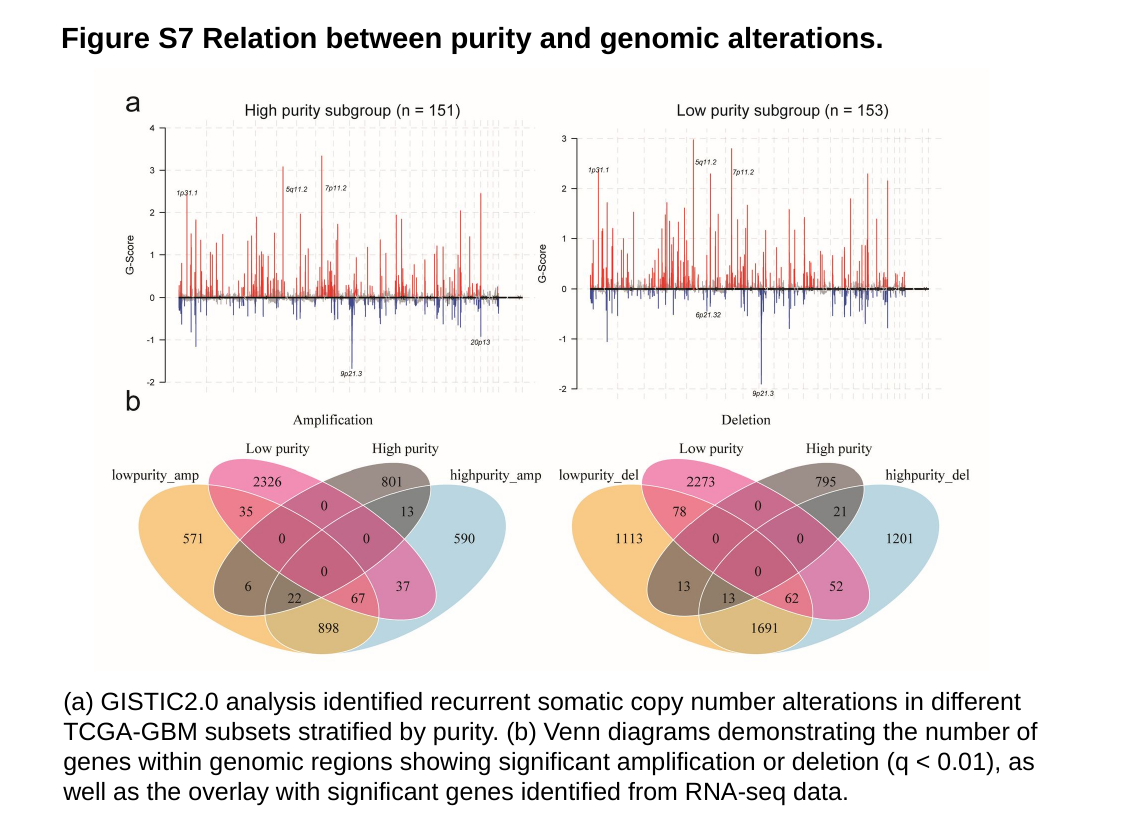

Figure S7 Relation between purity and genomic alterations.
(a) GISTIC2.0 analysis identified recurrent somatic copy number alterations in different TCGA-GBM subsets stratified by purity. (b) Venn diagrams demonstrating the number of genes within genomic regions showing significant amplification or deletion (q < 0.01), as well as the overlay with significant genes identified from RNA-seq data.

## Slide 8
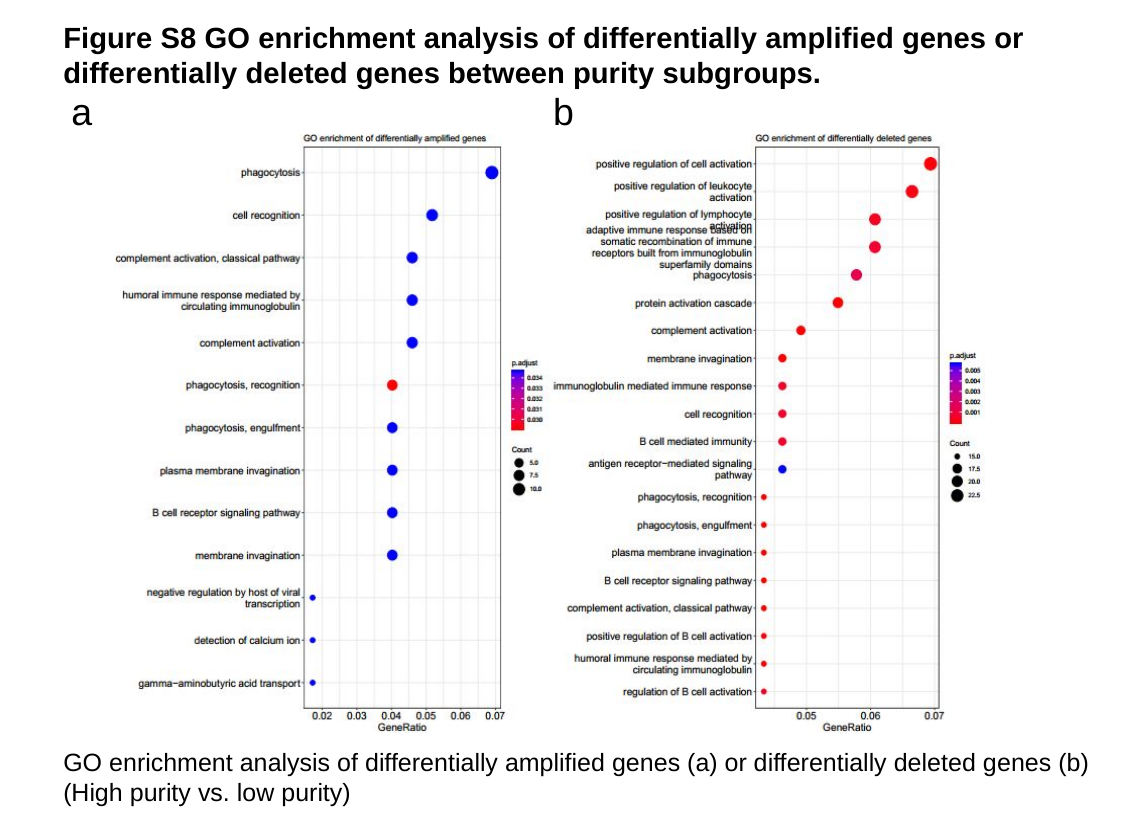

Figure S8 GO enrichment analysis of differentially amplified genes or differentially deleted genes between purity subgroups.
a
b
GO enrichment analysis of differentially amplified genes (a) or differentially deleted genes (b) (High purity vs. low purity)

## Slide 9
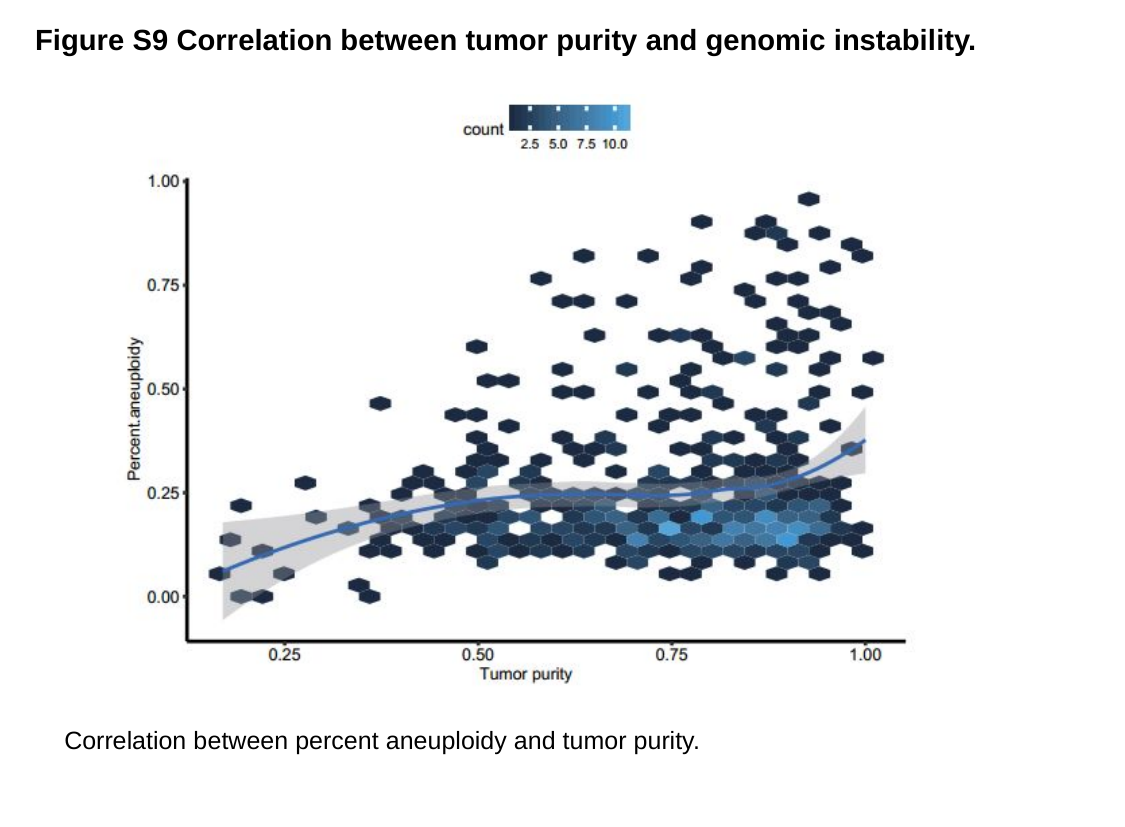

Figure S9 Correlation between tumor purity and genomic instability.
Correlation between percent aneuploidy and tumor purity.

## Slide 10
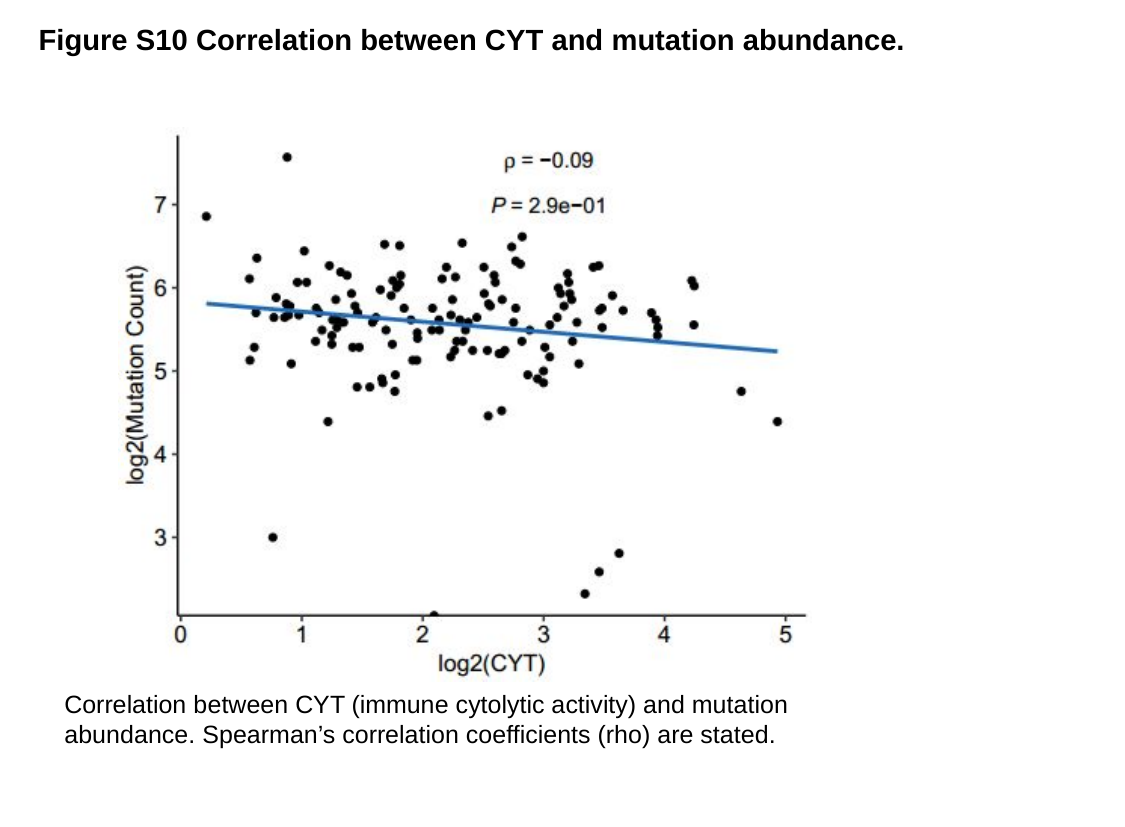

Figure S10 Correlation between CYT and mutation abundance.
Correlation between CYT (immune cytolytic activity) and mutation abundance. Spearman’s correlation coefficients (rho) are stated.
